# Supplementary material for: H2 ‐dependent modulation of tetrahydromethanopterin S‐methyltransferase (Mtr complex) activity by the small protein MtrR in Methanosarcina mazei
Source: FEBS J. 2026 Feb 13;293(14):4397–410. doi: 10.1111/febs.70457 (PMC13370731; doi:10.1111/febs.70457)
Supplement: Supplementary file 1 — Table S1. Proteins identified from Pulldown samples via LC–MS/MS. Table S2. Used Strains and plasmids. Table S3. Used Oligonucleotides. Fig. S1. Original files for Fig. 1: Purification and cellular localization of MtrR. Fig. S2. Original files for Fig. 2. MtrR interacts with the Mtr‐complex, particularly MtrA. Fig. S3. Southern Blot of the M. mazei WT and the DmtrR strain. [file FEBS-293-4397-s001.pdf]

Table S1: proteins identified from Pulldown samples via LC-MS/MS. In addition to the candidate proteins MtrA and MtrH, known contaminants trypsin (used for the digestion), and cytochrome C (used as a QC for the LC-MS system) were also identified in all samples

Sample ~26 kDa

Accession	Description	Coverage [%]	# Peptides	# PSMs	# Unique Peptides	# AAs	MW [kDa]	calc. pI	Score Sequest HT: Sequest HT	Replicates F2: Sample	F5: Sample	F8: Sample
O59640	Tetrahydromethanopterin S-methyltransferase subunit A OS=Methanosarcina mazei	32	6	23	6	240	**25.4**	5.45	49.63	High	High	High
P00004	Cytochrome c OS=Equus caballus	18	2	11	2	105	11.8	9.57	27.28	High	High	High
P00761	Trypsin OS=Sus scrofa	8	2	10	2	231	24.4	7.18	23.23	High	High	High

Control ~26 kDa

Accession	Description	Coverage [%]	# Peptides	# PSMs	# Unique Peptides	# AAs	MW [kDa]	calc. pI	Score Sequest HT: Sequest HT	F12: Sample	F15: Sample	F18: Sample
P00004	Cytochrome c OS=Equus caballus	18	2	14	2	105	11.8	9.57	35.57	High	High	High
P00761	Trypsin OS=Sus scrofa	16	3	9	3	231	24.4	7.18	20.12	High	High	High

Sample ~36 kDa

Accession	Description	Coverage [%]	# Peptides	# PSMs	# Unique Peptides	# AAs	MW [kDa]	calc. pI	Score Sequest HT: Sequest HT	F1: Sample	F4: Sample	F7: Sample
P80650	Tetrahydromethanopterin S-methyltransferase subunit H OS=Methanosarcina mazei	25	9	87	9	316	**34**	5.05	289	High	High	High
P00761	Trypsin OS=Sus scrofa	16	3	22	3	231	24.4	7.18	71.65	High	High	High
P00004	Cytochrome c OS=Equus caballus	18	2	6	2	105	11.8	9.57	14.23	High	High	High
Q60186	V-type ATP synthase alpha chain OS=Methanosarcina mazei	6	3	5	3	578	63.8	5.05	12.92	n/a	High	High
Q8PV47	Large ribosomal subunit protein uL2 OS=Methanosarcina mazei	11	2	3	2	238	25.7	10.13	8.74	High	High	n/a

Control ~36 kDa

Accession	Description	Coverage [%]	# Peptides	# PSMs	# Unique Peptides	# AAs	MW [kDa]	calc. pI	Score Sequest HT: Sequest HT	F11: Sample	F14: Sample	F19: Sample
P00004	Cytochrome c OS=Equus caballus	18	2	9	2	105	11.8	9.57	21.72	High	High	High
P00761	Trypsin OS=Sus scrofa	16	3	5	3	231	24.4	7.18	11.75	n/a	High	High

Table S2: Used Strains and plasmids

**Archaea**		
***Methanosarcina mazei* Goe1**	Wildtype	German Collection of Microorganisms and Cell Cultures GmbH (DSMZ) number 3647
***Methanosarcina mazei* wt (3A)**	Wildtype,<br>improved growth on solid media	(Ehlers *et al.*, 2005)
***Methanosarcina mazei* ΔsORF16**	*M. mazei* 3A/ΔsP36 genomic deletion	This work
**Bacteria**		
***E.* coli DH5α**	host strain for cloning of plasmid constructs	(Hanahan, 1983)
***E.* coli BL21 (DE3) contianing pRIL**	strain for overexpression of genes with unusual codon usage; Cm^R	Stratagene, La Jolla, USA
***E. coli* C43 (DE3) containing pRIL**	strain for overexpression of hydrophobic or toxic proteins with unusual codon usage; Cm^R	(Miroux and Walker, 1996)
**Plasmids**		
**pmcl210**	cloning Vector, P15Aori, *lacZ*, *cat*	(Nakano *et al.*, 1995)
**pRIL**	Encoding additional tRNAs	Stratagene, La Jolla, USA
**pET21a(+)**	expression vector, His-tag, Amp^R	Novagen, Darmstadt, Germany
**pRS1736**	expression vector, strep-tag, Amp^R	Tristan et al., 2025
**pRS207**	pac cassette in pBlueskript SK	(Ehlers *et al.*, 2005)
**pRS1639**	*pMcrB;*His_6*-MMsORF16,* Pur^R*,*	(Tufail *et al.*, 2024)
**pRS1743**	*Twin-Strep*-MM_1547, Pur^R	(Reif-Trauttmansdorff *et al.*, 2025)
**pRS1834**	*pET21a/MMsORF16,* Amp^R	This work
**pRS2037**	Pmcl210/MMsORF16, flanking regions, Pur^R	This work
**pRS2087**	His_6*-MMsORF16,* Pur^R	(Tufail *et al.*, 2024)
**pRS2190**	*pRS1736/MM1543* (aa 1-168)*,* Amp^R	This work

Table S3: Used Oligonucleotides

construct	primer	Sequence 5'→ 3'
Δ*mtrR*	sORF16+1kb_for	TTTGCGGCCGCCTCCATCATAGTTGAGCTACCG
	sORF16+1kb_rev	TTTGGATCCGGAACAGCTGTCTCCCGC
	sORF16-1kb_for	TTTGGATCCGGCTCACCTTTGATTGAACTTTTG
	sORF16-1kb_rev	TTTGGTACCCTTTAAAATGGGGATTGCAGGTG
mtrR-His_6 / *mtrR*-probe	sORF16_nde_for	TTTCATATGAAAGAAATTATTACGCAAAAGCCTCTG
	sORF16_not1_rev	TTTGCGGCCGCGGTCTCCTCCGACTTTATG
strep-*mtrA*	mtrA_nde1_fwd	CATATGGCAGATAAAAGAGAACCAGC
	mtrA_not1_rev	GCGGCCGCTTACCTCCCTAGCAGTAATAACC

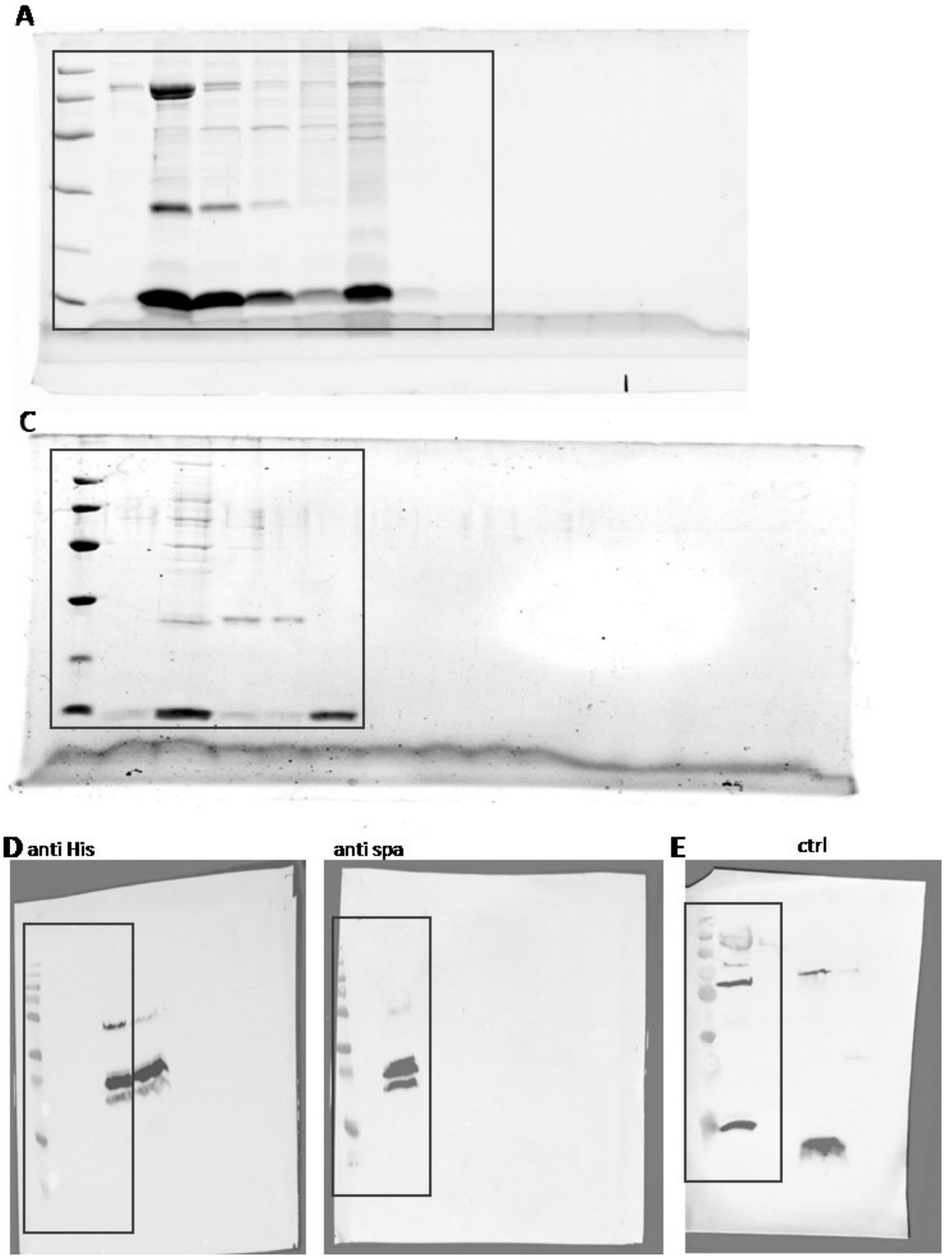

Figure S1: Original files for Figure 1: Purification and cellular localization of MtrR. For all images the frame indicates the part, used for the publication figure. A: SDS PAGE of IMAC purification of heterologously expressed MtrR-His$_6$. **B:** SEC elution fractions on SDS PAGE; depicted are fractions 09 - 13 ml. **C:** Exponentially growing *M. mazei* cells expressing MtrR-His$_6$ or spa-MtrR from plasmids pRS2087 and pRS1693, respectively, were harvested, fractionated, and subcellular fractions analyzed by western blot using antibodies targeting either the His- or spa-tag. **E:** *In vivo* copurification: MtrR-His$_6$ was constitutively expressed in *M. mazei* (pRS2087) and purified from the solubilized membrane fraction (2 % DDM) by IMAC. Elution fraction two of the purified MtrR complex was analyzed by western blot using antibodies generated against MtrR. The additional band visible on the gel, which was cut, corresponds to purified MtrR as an internal control to assure the success of the western blot.

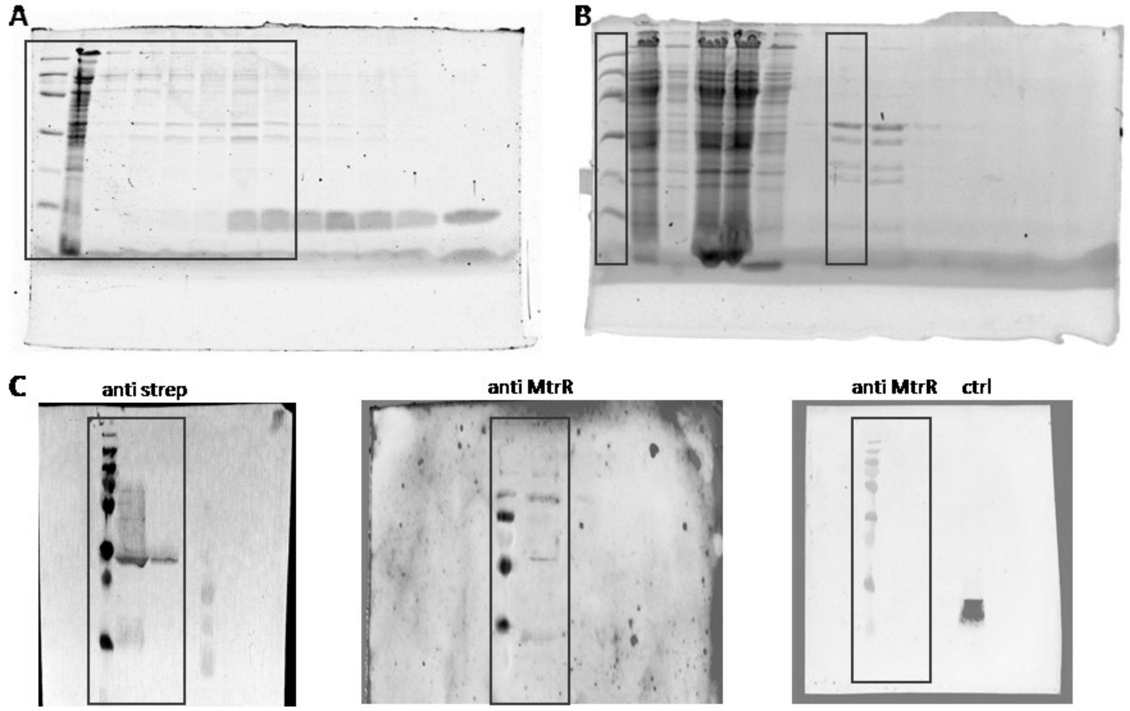

Figure S2: Original files for Figure 2. MtrR interacts with the Mtr-complex, particularly MtrA. For all images the frame indicates the part, used for the publication figure. A: SDS-PAGE resulting from pull-down analyses using purified MtrR-His$_6$ as bait against solubilized *M. mazei* membrane fractions (2 % DDM). After 30 min incubation at RT, MtrR-His$_6$ was purified by IMAC revealing two additional distinct protein bands. **B and C:** *In vivo* co-purification of the Mtr complex from *M. mazei* using plasmid encoded strep-MtrE. **B**: Elutions of the strep-tactin purified complex were analyzed by SDS PAGE. Visible are the different copurified subunits of the Mtr complex. Several bands were cut from the gel which correspond to different steps during purification as internal controls. from left to right: whole cell extract, unlysed cell fraction, solubilized membrane fraction, flow through, first washing step, second washing step. **C:** Elutions of the strep-tactin purified complex were analyzed via western blot using antibodies directed against the strep-tag or MtrR. *M. mazei* WT cells not encoding any strep tagged proteins were used as a control (right panel); The single band visible on the gel which was cut, corresponds to purified MtrR, used as an internal control to assure the success of the western blot.

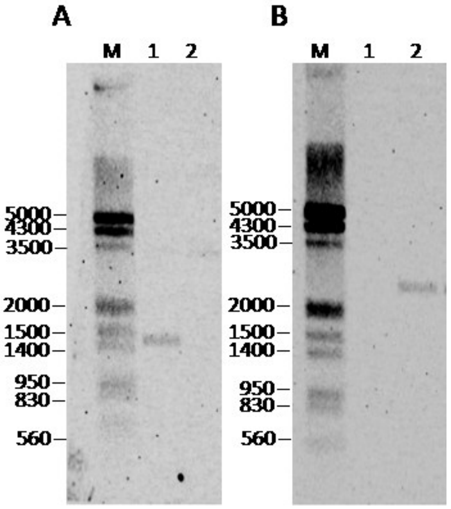

Fig. S3: Southern Blot of the *M. mazei* WT (1) and the D*mtrR* strain (2) with specific probes targeting the *sORF16* gene (A) or the puromycin resistance cassette (B). The *mtrR* is only present in the WT, while it has been exchanged with the pac in the D*mtrR* strain confirming the successful genomic deletion of *mtrR*. M: DNA Molecular Weight Marker III DIG-labeled, 0.12.21.2 kbp (Roche, Basel, Switzerland)
